# Deciphering the Genetic Alteration in the ZEB2 Gene Network and Their Possible Association With Head and Neck Squamous Cell Carcinoma (HNSCC)

**DOI:** 10.7759/cureus.46440

**Published:** 2023-10-03

**Authors:** Dhivya S, Anitha P, Smiline Girija AS, Paramasivam A, Vijayashree Priyadharsini J

**Affiliations:** 1 Microbiology, Saveetha Dental College and Hospitals, Saveetha Institute of Medical and Technical Sciences, Saveetha University, Chennai, IND; 2 Clinical Genetics Lab, Centre for Cellular and Molecular Research, Saveetha Dental College and Hospitals, Saveetha Institute of Medical and Technical Sciences, Saveetha University, Chennai, IND; 3 Molecular Biology Lab, Centre for Cellular and Molecular Research, Saveetha Dental College and Hospitals, Saveetha Institute of Medical and Technical Sciences, Saveetha University, Chennai, IND

**Keywords:** survival analysis, gene expression profile, oral cancers, somatic mutations, human population genetics

## Abstract

Background

Head and neck squamous cell carcinoma (HNSCC) is an abnormal growth of cells that leads to tumor formation in the head and neck region. Several genes and genetic networks are involved in the process of carcinogenesis.

Aim

The aim of the present study is to unravel the prognostic marker from a pool of interacting networks governed by the ZEB2gene*.*

Materials and methods

Computational analysis was employed to identify the protein network interactions, genetic alterations, gene expression, and the survival analysis of the ZEB2 dysregulated network in the head and neck cancer dataset (HNSCC) from the Cancer Genome Atlas (TCGA), Firehose Legacy. The gene expression profiling and survival analysis were performed for the gene with the highest frequency of genetic alteration.

Result

The interaction network returned nine genes that interact with ZEB2. The ARHGAP31 gene was found to harbor the highest frequency of alteration at the genomic as well as the transcriptomic levels. Survival was also found to be significant with respect to the differential gene expression pattern while comparing the genders and different ethnic groups. Females with higher expression of ARHGAP31 and the Asian population exhibiting low/medium expression of the same were found to present with poor survival probability.

Conclusion

The identification of putative drivers or a candidate gene of a network could provide clues about the association with the disease phenotype of HNSCC. The present study identifies ARHGAP31 as the key gene of the ZEB2 gene network, wherein the genetic alterations correlate with the transcriptomics data and the survival probability of patients segregated based on gender and race. Further experimental evaluation is warranted to confirm the association of this infamous gene ARHGAP31 with the development of oral carcinoma.

## Introduction

Head and neck squamous cell carcinoma (HNSCC) refers to a type of cancer that arises in the cells that line the mucosal surfaces of the head and neck region, including the mouth, throat, voice box, sinuses, and salivary glands [1]. The GLOBOCAN 2020 report estimates that in 2020, lip and oral cavity cancer would be responsible for about 355,000 new cases and 177,000 fatalities worldwide. The incidence rate of lip and oral cavity cancer varies greatly by geographic location, with South Asia and Southeast Asia having the highest rates. In contrast, Europe and North America have the lowest incidence rates. Two of the major risk factors for developing lip and oral cavity cancer are alcohol consumption and tobacco use. Poor oral hygiene, human papillomavirus (HPV) infection, and sun exposure are other risk factors. Depending on the stage and location of the disease, surgery, radiation therapy, and/or chemotherapy are frequently used as treatments for lip and oral cavity cancer [2]. Early diagnosis and treatment can significantly increase the likelihood of survival. For patients with HNSCC, early identification and therapy are essential for improving outcomes. Frequent dental and medical exams, limiting or quitting alcohol and tobacco use, and regular dental and medical checkups can all help reduce the chance of having this kind of cancer [3].

ZEB2 (zinc finger E-box binding homeobox 2) is a transcription factor that plays a crucial role in embryonic development and differentiation. However, it is also involved in the development and progression of various types of cancer. Many forms of cancer, including breast [4], lung [5], ovarian [6], colorectal [7], gastric [8], and pancreatic cancers [9], have been linked to an upregulation of ZEB2. Epithelial-mesenchymal transition (EMT), a process through which epithelial cells lose their cell-cell adhesion and acquire a mesenchymal phenotype with greater motility and invasiveness, has been demonstrated to promote cancer cell migration, invasion, and metastasis [10]. The ZEB2 gene has been linked to the promotion of cancer stem cell qualities, chemoresistance, and immune evasion in several forms of cancer in addition to its role in promoting invasion and metastasis of cancer cells [4]. The role of ZEB2 in cancer appears to be complex and multifaceted overall, and it probably varies based on the particular cancer type and environment. To create novel cancer therapies, however, targeting ZEB2 or its downstream signalling pathways may be a potential approach.

## Materials and methods

Protein-protein interaction network analysis

STRING is a database and web resource that provides information about protein-protein interactions, as well as other types of functional associations between proteins, such as shared pathways and co-expression patterns. The interactions and associations in STRING are derived from a variety of sources, including experimental data, computational predictions, and literature mining. The STRING is a valuable resource for researchers to analyze protein function, signalling pathways, and disease mechanisms. It can help researchers identify new protein-protein interactions, predict the functions of uncharacterized proteins, and generate hypotheses about the roles of proteins in cellular processes [11]. This resource was employed to identify the interaction network of the ZEB2 gene in the present study.

Sample data sets

The sample data for the study were retrieved from the cBioportal database [12,13]. The database hosts more than 300 cancer research on the open-access portal known as cBioPortal for the interactive exploration of multidimensional cancer genomics data sets. The platform enables scientists to query and visualize different kinds of genomic data, including somatic mutations, mRNA expression, and DNA copy number alterations, and to examine the connections between various genes and their clinical effects. cBioPortal is an effective tool for cancer genomics research that enables investigators to look into the genomic basis of cancer and find prospective treatment targets. The HNSCC dataset (TCGA, Firehose Legacy) provides information on 530 samples, of which the mutation and copy number alteration data are available for 504 samples. These data were further used for analysis (Table 1).

**Table 1:** Demographic details of patients analyzed in the present study (as obtained from the cBioportal site)

Variable	Number
Gender	
Male	386
Female	142
Mutation count	6-3181
Diagnosis age	19-90 years
Smoking status	
Smokers	515
Data not available	12
Unknown	1
Alcohol history	
Yes	352
No	165
Data not available	11
Neoplasm Histologic grade	
Grade 1	63
Grade 2	311
Grade 3	125
Grade 4	7
Grade GX	18
Data not available	4
Race category	
White	452
African	48
Asian	11
American Indian or Alaska Native	2
Data not available	15

Oncoprint analysis

Oncoprint is a sort of data visualization that's used to show how cancer's genetic changes are represented. It is a frequently used method in cancer genomics research and is widely utilized to present complex datasets in a condensed, coherent form. Oncoprints display multiple genomic alterations in a single patient or across a cohort of patients. Several types of genomic alterations, such as mutations, amplifications, deletions, and fusions, are represented by different symbols or colours. The presence of an alteration is indicated by a filled-in symbol, and the absence of an alteration is represented by a grey bar [12,13].

Gene expression and survival analysis

UALCAN (the University of ALabama at Birmingham CANcer data analysis Portal) is a web-based platform that provides researchers with easy and interactive access to cancer transcriptome and proteomic data from the Cancer Genome Atlas (TCGA) database. UALCAN enables users to analyze gene expression levels, clinical data, and survival analysis across various cancer types, including breast, lung, liver, colon, and ovarian cancer. This computational tool was used to reveal the gene expression profile and the concomitant survival status of HNSCC patients in comparison to normal tissue samples [14].

## Results

Oncoprint data analysis

**Figure 1:**
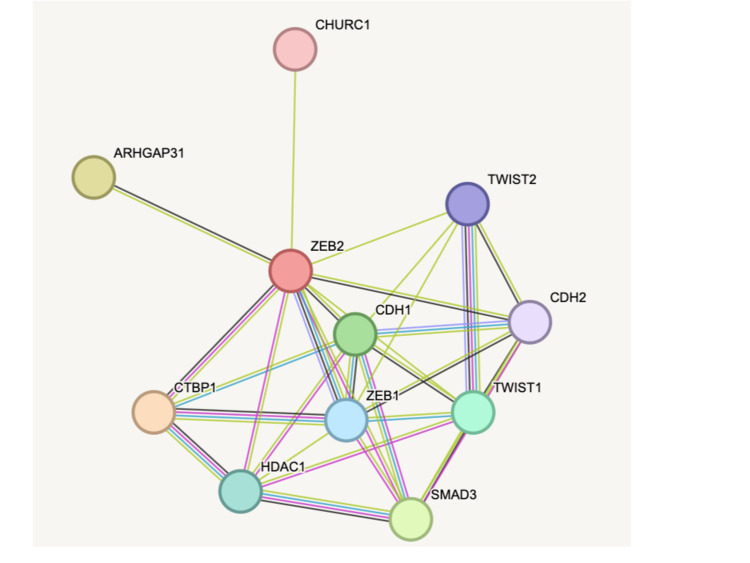
The protein interaction network of the ZEB2 gene demonstrating its primary interaction with nine genes. The interactions were either experimentally determined (pink), co-occurence of genes (blue), or text-mining (green).

**Figure 2:**
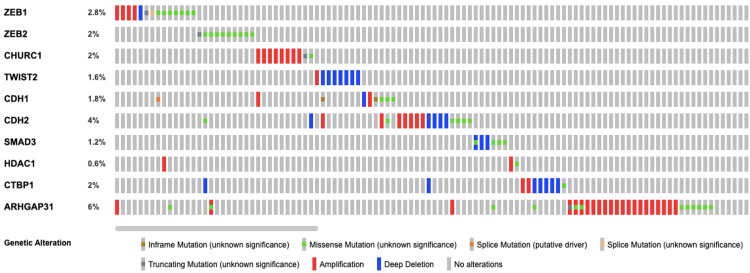
Oncoprint analysis depicting gene alterations in the ZEB2 interacting genes. Each of the grey bars represents patients with head and neck squamous cell carcinoma.

The analysis of the oncoprint data indicated changes in 10 genes viz., ZEB1, ZEB2, CHURC1, TWIST2, CDH1, CH2, SMAD3, HDAC1, CTBP1, ARHGAP31 (Figure 1). Among all the genes, the frequency of ARHGAP31 was found to be the 6% highest among all the other genes in the network (Figure 2). Gene amplification was the predominant form of alteration with a few missense and truncating mutations in this gene. Apart from this splice site mutations, deletions, and inframe mutations were also found in other genes analyzed. The mutation profiles were highly unique and the possibility of one patient harbouring multiple genetic alterations pertaining to the ZEB2gene network was low.

Gene expression and survival analysis

**Figure 3:**
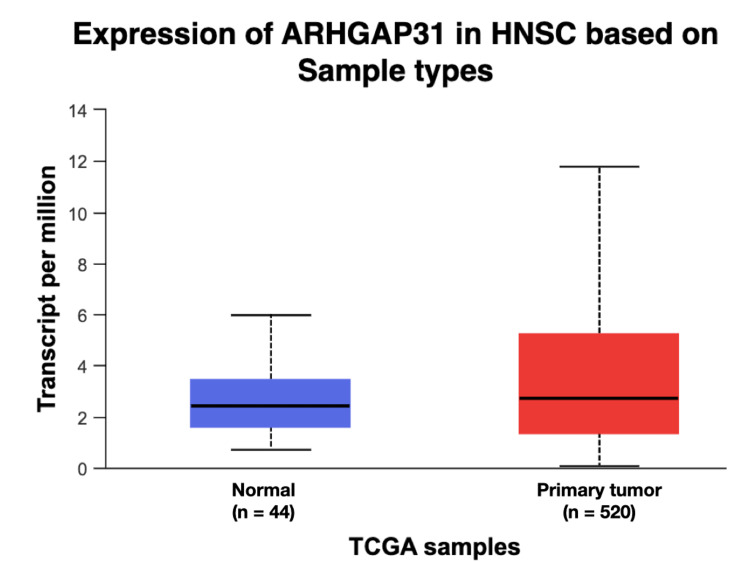
Box whisker plot demonstrating differential gene expression pattern in head and neck squamous cell carcinoma patients when compared to normal samples. The ARHGAP31 gene was found to exhibit a significant difference between the two groups with a p-value of 3.75 x 10-2. A p-value less than 0.05 is considered to be significant.

**Figure 4:**
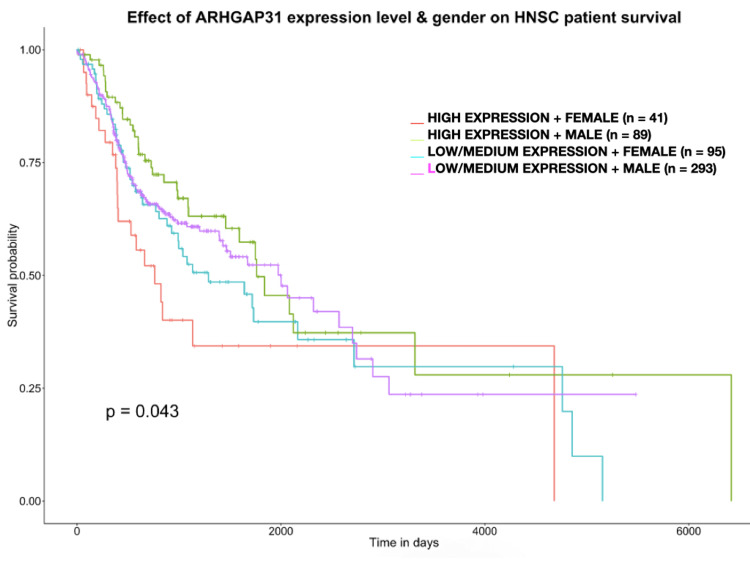
Kaplan-Meier survival plot demonstrating the ARHGAP31 gene expression levels based on the gender of HNSCC patients and their influence on patient’s survival. High expression of ARHGAP31 was found to affect the survival probability to a significant extent (p-value = 0.043) when compared to levels of expression in other gender.

**Figure 5:**
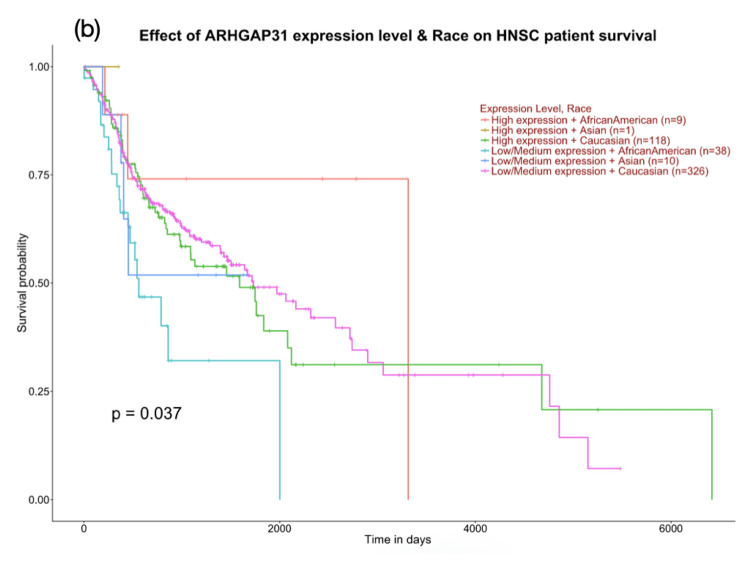
Kaplan-Meier survival plot demonstrating the ARHGAP31 gene expression levels based on the gender of HNSCC patients and their influence on patient’s survival. Low expression of ARHGAP31 in the Asian population was found to be associated with poor survival probability. A p-value less than 0.05 was found to be significant.

The gene expression data acquired from the UALCAN database revealed a significant difference in the expression of ARHGAP31. This change was found to be in agreement with the genetic alteration. A p-value less than 0.05 is considered to be significant. The maximum TPM (transcript per million) value was found to be 11.794 while the minimum was found to be 0.088 in the tumour group, whilst the maximum and minimum values were found to be 5.9 and 0.73 respectively, creating a marked difference in the gene expression profile (p = 3.75 x 10-2) (Figure 3). The Kaplan-Meier survival analysis also revealed a significant difference between the levels of ARHGAP31 expression among males and females. The female subjects expressing a high level of ARHGAP31 experienced a poor survival probability when compared to the male subjects with high, low/medium expression of ARHGAP31. Also, low/medium expression levels of ARHGAP31 were found to be associated with poor survival probability in Asian HNSCC patients. The expression profile is largely influenced by epigenetic factors such as DNA methylation, histone modification and modifications to the transcripts exerted by non-coding RNAs on the target gene (Figures 4-5). Hence, exploring the epigenetic profile, and investigating the gender and ethnic variabilities contributing to the differential expression might provide vital clues on the association of ARHGAP31’s role in oral carcinogenesis.

## Discussion

Cancer is a complex multifactorial, multigenic disorder, that requires careful assessment of not only the candidate genes but also their interacting networks. Numerous studies providing preliminary data on candidate genes associated with oral cancer and their prognostic significance have been dealt with earlier. The antioxidant genes [15], methylation markers [16-18], cancer gene networks [19], epithelial-mesenchymal markers [20], xenobiotic metabolizing enzyme encoding genes [21], and many more have been investigated to derive potential clues about the putative drivers of oral cancer. In line with this concept, the present study was designed to identify the candidate gene of the ZEB2 gene network, that can be utilized as a potential prognostic and diagnostic marker.

The transcription factor ZEB2 (also known as SIP1) is involved in tissue homeostasis and embryonic development. It also plays a role in controlling the epithelial-mesenchymal transition (EMT), which is the process by which epithelial cells lose their polarity and adhesion and develop a mesenchymal phenotype, enabling them to move and invade neighbouring tissues. Arunkumar and the team provided evidence that ZEB2may play a role in the initiation and spread of oral cancer. The expression of ZEB2 was shown to be considerably higher in oral squamous cell carcinoma (OSCC) tissues compared to healthy oral tissues. The study also discovered that among OSCC patients, increased ZEB2 expression was linked to both a worse prognosis and a higher probability of lymph node metastases [22]. Kim and colleagues discovered that OSCC cells that had undergone EMT had higher ZEB2 expression, which was linked to greater cell motility and invasion. Overall, these results indicated that ZEB2, presumably through its involvement in EMT and cell migration/invasion, contributed to the initiation and spread of oral cancer [23].

ZEB2 has been identified as one of the key controllers of the epithelial-mesenchymal transition (EMT) process, which is important in the development and spread of cancer. Oral cancer stem cells (OCSCs), a subpopulation of cancer cells found in OSCC, are thought to be crucial for the development, growth, and recurrence of tumours. Recent research has documented that ZEB2 may have a role in the control of OCSCs. For instance, a study by Chu et al. discovered that OCSCs had more ZEB2 expression than non-stem cancer cells in OSCC and that ZEB2 knockdown decreased the self-renewal and tumorigenicity of OCSCs. Moreover, a different study discovered that OSCC patients with high ZEB2 expression had a bad prognosis, while ZEB2 knockdown did not. Taken together, these findings suggest that ZEB2 may play an important role in the regulation of OCSCs in OSCC. Further research is needed to fully understand the mechanisms by which ZEB2 regulates OCSCs and to determine whether targeting ZEB2 could be a viable strategy for the prevention of OSCC recurrence [24]. Several non-coding RNAs were found to target ZEB1 and ZEB2 translation in various cancer types. A study conducted to understand the interaction of Kindlin-2 and ZEB2 with their common microRNA target miR-200b revealed upregulation of ZEB2 and Kindlin-2 and downregulation of miR-200b, thereby facilitating the invasion and migratory potential of OSCC cells [25].

The key gene identified from the ZEB2 gene network of the present study was the ARHGAP31 (also known as RHG31) which encodes for a protein called Rho GTPase-activating protein 31, which plays a role in the regulation of cell movement and division. Mutations in this gene have been associated with various types of cancer, including oral cancer. Rho GTPases are enzymes that regulate cell migration and invasion, and studies have shown that mutations in the ARHGAP31 gene may affect their activity, which may contribute to the onset and spread of oral cancer. Particularly, mutations in the ARHGAP31 gene could result in enhanced RhoA GTPase activity, which might encourage tumour development and metastasis. It is crucial to keep in mind that oral cancer is a complicated condition that is influenced by a number of genetic and environmental factors and that the ARHGAP31 gene's function in this process is currently being investigated [26].

As with any other procedure, the in silico analysis has certain limitations. Firstly, the population for which the analysis is performed belongs largely to the American and African ethnic groups. Secondly, habits, environmental and lifestyle factors have a profound role to play in the initiation and progression of cancer, which might have a greater influence on the genetic alterations. Thirdly, only the preliminary network encompassing the 10 proteins interacting with ZEB2 was analyzed; it will be worthwhile to investigate other networks or an extended network of genes interacting with ZEB2. Taking into consideration all these limitations, population-based mutation and expression profiling will provide concrete evidence of the possible association of ZEB2 with oral cancer. Therefore, novel genes identified from the gene networks serve as an excellent source for developing test panels. The differentially expressed genes with unique correlations to a specific population are sure to enhance the diagnostic as well as prognostic procedures [27].

## Conclusions

Studies precisely designed based on the interaction networks can more appropriately aid in the selection of crucial targets for developing diagnostic panels. The present study throws light on the interaction network of a crucial gene involved in various hallmarks of cancer, with a special emphasis on the EMT process. As cancer is a polygenic disorder, the selection of markers based on the interaction network of key genes could provide more insights into the pathways leading to cancer. Taken together, the present study revealed ARHGAP31 as a vital target in the ZEB2 gene network, as it was shown to have profound effects on the survival of HNSCC patients. Thus, future studies may be directed towards understanding the relationship between ZEB2 and ARHGAP31 proteins and their functions in the process of initiating treatment of tumours by performing functional analysis. Furthermore, validation of ARHGAP31 to serve as a diagnostic, prognostic or therapeutic target is warranted to achieve success with the management of HNSCC.
